# Treatment of multiple traumatized adolescents by enhancing regulation skills and reducing trauma related symptoms: rationale, study design, and methods of randomized controlled trial (the Mars-study)

**DOI:** 10.1186/s12888-023-05073-4

**Published:** 2023-09-04

**Authors:** Rik Knipschild, Helen Klip, Doenja van Leeuwaarden, Mariken J. R. van Onna, Ramon J. L. Lindauer, Wouter G. Staal, Iva A. E. Bicanic, Ad de Jongh

**Affiliations:** 1 Child and Adolescent Psychiatry, Nijmegen, The Netherlands; 2 Levvel Academic Center for Child and Adolescent Psychiatry, Amsterdam, The Netherlands; 3 Department of Child and Adolescent Psychiatry, Amsterdam UMC, Location AMC, University of Amsterdam, Amsterdam, The Netherlands; 4 Department of Psychiatry, Radboud University Medical Centre, Nijmegen, The Netherlands; 5 Leiden Institution for Brain and Cognition, Leiden, The Netherlands; 6 National Psychotrauma Centre for Children and Youth, University Medical Centre Utrecht, Utrecht, The Netherlands; 7 Academic Centre for Dentistry Amsterdam (ACTA), University of Amsterdam and VU University Amsterdam, Amsterdam, Netherlands; 8 Psychotrauma Expertise Centre (PSYTREC), Bilthoven, Netherlands; 9 School of Health Sciences, Salford University, Manchester, UK; 10 Institute of Health and Society, University of Worcester, Worcester, UK; 11 School of Psychology, Queen’s University, Belfast, Northern Ireland

**Keywords:** Trauma, PTSD, Complex PTSD, Adolescents, EMDR, STAIR, TRAP, Stabilization, Phase-based treatment

## Abstract

**Background:**

There is ongoing debate regarding the treatment of severe and multiple traumatized children and adolescents with post-traumatic stress disorder (PTSD). Many clinicians favor a phase-based treatment approach (i.e., a stabilization phase prior to trauma-focused therapy) over immediate trauma-focused psychological treatment, despite the lack of scientific evidence. Research on the effects of different treatment approaches is needed for children and adolescents with (symptoms of complex) PTSD resulting from repeated sexual and/or physical abuse during childhood.

**Objective:**

This paper describes the rationale, study design, and methods of the MARS-study, a two-arm randomized controlled trial (RCT) that aims to compare the results of phase-based treatment with those of immediate trauma-focused treatment and determine whether immediate trauma-focused treatment is not worse than phase-based treatment in reducing PTSD symptoms.

**Methods:**

Participants are individuals between 12 and 18 years who meet the diagnostic criteria for PTSD due to repeated sexual abuse, physical abuse, or domestic violence during childhood. Participants will be blindly allocated to either the phase-based or immediate trauma-focused treatment condition. In the phase-based treatment condition, participants receive 12 sessions of the Dutch version of Skill Training in Affective and Interpersonal Regulation (STAIR-A), followed by 12 sessions of EMDR therapy. In the immediate trauma-focused condition, the participants receive 12 sessions of EMDR therapy. The two groups are compared for several outcome variables before treatment, mid-treatment (only in the phase-based treatment condition), after 12 trauma-focused treatment sessions (post-treatment), and six months post-treatment (follow-up). The main parameter is the presence and severity of PTSD symptoms (Clinician-Administered PTSD Scale for Children and Adolescents, CAPS-CA). The secondary outcome variables are the severity of complex PTSD symptoms (Interpersonal Problems as measured by the Experiences in Close Relationship-Revised, ECR-RC; Emotion Regulation as measured by the Difficulties in Emotion Regulation Scale, DERS; Self Esteem as measured by the Rosenberg Self Esteem Scale, RSES), changes in anxiety and mood symptoms (Revised Anxiety and Depression Scale; RCADS), changes in posttraumatic cognitions (Child Posttraumatic Cognitions Inventory, CPTCI), changes in general psychopathology symptoms (Child Behavior Checklist, CBCL), and Quality of Life (Youth Outcome Questionnaire, Y-OQ-30). Furthermore, parental stress (Opvoedingsvragenlijst, OBVL) and patient-therapist relationship (Feedback Informed Treatment, FIT) will be measured, whereas PTSD symptoms will be monitored in each session during both treatment conditions (Children’s Revised Impact of Event Scale, CRIES-13).

**Discussion:**

Treating (symptoms of complex) PTSD in children and adolescents with a history of repeated sexual and/or physical abuse during childhood is of great importance. However, there is a lack of consensus among trauma experts regarding the optimal treatment approach. The results of the current study may have important implications for selecting effective treatment options for clinicians working with children and adolescents who experience the effects of exposure to multiple interpersonal traumatic events during childhood.

**Trial registrations:**

The study was registered on the “National Trial Register (NTR)” with the number NTR7024. This registry was obtained from the International Clinical Trial Registry Platform (ICTRP) and can be accessed through the ICTRP Search Portal (https://trialsearch.who.int/).

## Background

A meta-analysis of children and adolescents exposed to traumatic events indicated that 16% developed post-traumatic stress disorder (PTSD) [1]. Adolescents with PTSD re-experience traumatic events, avoid memories of the trauma, develop negative thoughts and moods, and are hypervigilant about potential threats [2]. Adolescents who have been exposed to (repeated) sexual abuse, maltreatment, and/or domestic violence are at an even higher risk of developing PTSD symptoms [1]. Beyond the core symptoms of PTSD, adolescents are prone to developing a range of additional challenges, including poor self-esteem, difficulties in interpersonal relationships, and struggles with emotion regulation. Collectively, these symptoms are commonly referred to as characteristics of the ICD-11 classification Complex PTSD [3, 4].

Although unprocessed trauma can significantly impact a child's development, several studies have demonstrated the effectiveness of trauma-focused treatments, such as eye movement desensitization and reprocessing (EMDR) therapy, trauma-focused cognitive behavioral therapy (TF-CBT), and Prolonged Exposure [5, 6], in treating childhood PTSD. As a result, trauma-focused treatments are generally recommended in national guidelines for children and adolescents [7] and international guidelines from reputable organizations, such as the International Society for Traumatic Stress Studies [8] and the National Institute for Health and Clinical Excellence [9]. However, it is worth noting that most studies underlying these guidelines have primarily focused on children and adolescents who developed PTSD due to a single traumatic event, leaving a knowledge gap regarding the efficacy of trauma-focused therapies for children and adolescents with Complex PTSD and a lack of consensus on the preferred treatment approaches for this population.

A position paper by the International Society of Traumatic Stress Studies [10] highlighted the insufficient evidence available to support a specific treatment for Complex PTSD in children and adolescents. This dearth of evidence has sparked long-standing debate among clinicians and researchers regarding the optimal approach for treating Complex PTSD. Recognizing the potential limitations of existing evidence-based treatments for children and adolescents with Complex PTSD and severe clinical presentations, the ISTSS argues that modifications to trauma-focused treatments may be necessary to address the symptoms of Complex PTSD effectively [10]. Consequently, various phase-based treatment models have been developed for children and adolescents with Complex PTSD [11, 12]. These models generally prioritize the enhancement of emotional and interpersonal regulation skills as well as strengthening self-esteem before embarking on trauma-focused treatment. However, it is important to note that a comprehensive body of compelling evidence supporting the efficacy of phase-based models for the treatment of Complex PTSD is lacking [13]. Critics of phase-based models state that research on adults with Complex PTSD has shown that the incorporation of regulation skills training prior to trauma-focused therapy unnecessarily prolongs therapy [14–16]. In addition, studies using trauma-focused treatment in adolescents have demonstrated promising results in the treatment of PTSD and Complex PTSD with a history of (repeated) sexual abuse and maltreatment [17–20], albeit with inconsistent findings and variations in the adaptation of treatment models.

Hence, the limited number of studies investigating the effectiveness of treatments for young individuals with Complex PTSD underscores the need for further research [10]. In their position paper published in 2019, the ISTSS deemed treatment recommendations premature because of current knowledge gaps. This indicates a pressing requirement for research exploring the effects of trauma-focused treatment on adolescents with (symptoms of complex) PTSD resulting from (repeated) sexual abuse, maltreatment, and/or domestic violence. To address this research gap, the present study describes an RCT aimed at investigating the differences in treatment effects between two treatment approaches (i.e., phase-based versus immediate trauma-focused) in adolescents with (symptoms of complex) PTSD resulting from a history of repeated sexual and/or physical abuse during childhood.

The primary objective of this study is to compare the results of phase-based treatment with those of immediate trauma-focused (i.e., eye movement desensitization and reprocessing; EMDR) therapy using a two-arm randomized controlled trial design. The first aim is to determine whether immediate trauma-focused treatment is not worse than phase-based treatment in reducing PTSD symptoms. If trauma-focused treatment is found to be equally effective when applied, it could lead to significant reductions in treatment duration. Second, we aim to investigate whether the phase-based therapy approach yields superior outcomes compared to the direct trauma-focused condition in terms of Complex PTSD symptoms, including emotion regulation, interpersonal problems, and self-esteem. Additionally, we will examine comorbid symptoms and dropout rates as secondary outcome measures. The third objective of the current study is to identify potential moderators and predictors of dropout or treatment responses/non-responses under both treatment conditions. We hypothesize that the presence of affect dysregulation and interpersonal problems at the beginning of therapy will be associated with poorer outcomes in the direct trauma-focused condition. Finally, we will examine the relationship between the reduction of posttraumatic stress symptoms in adolescents and a decrease in self-reported parental/caretaker stress. This objective addresses the clinical assumption that managing parental stress should be prioritized before initiating PTSD treatment in adolescents.

## Method

### Study design

**Fig. 1:**
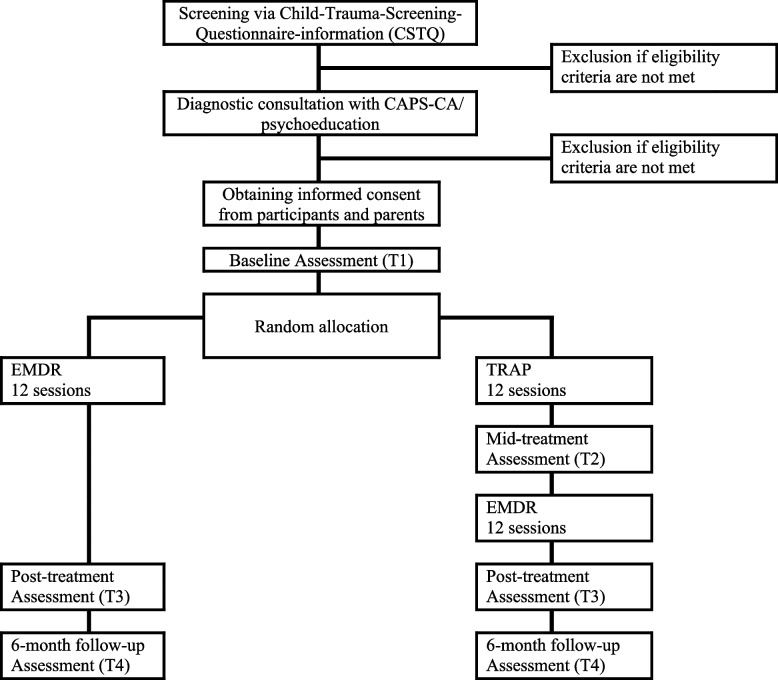
Flowchart of the MARS-study

This study entails a single-blind, randomized controlled trial with two arms: a phase-based treatment condition (TRAP followed by EMDR) and a trauma-focused treatment condition (EMDR only). In the TRAP-EMDR condition, participants receive 12 sessions of skills training (TRAP; the Dutch adolescents version of the STAIRS protocol), followed by 12 sessions of EMDR therapy. In the other condition, the participants receive 12 EMDR sessions. The two groups will be compared for several outcome variables before treatment, immediately after 12 sessions (post-treatment), and six months post-treatment (follow-up) (see Fig. 1). This study will be coordinated by the Karakter Academic Center for Child and Adolescent Psychiatry in the Netherlands. The study started in 2018, paused for seven months in 2020 due to COVID-19, and continued in September 2020. This study is ongoing and is expected to be completed by 2024.

### Participants

Participants are individuals between 12 and 18 years of age, meeting the criteria for PTSD (according to the Diagnostic and Statistical Manual of Mental Disorders, fifth edition), and victims of repeated sexual and/or physical abuse and/or domestic violence in childhood. Participants are recruited from different departments of Karakter, a large mental health organization for children and adolescents in the Netherlands. This applies the following inclusion criteria: (1) meeting the criteria for PTSD, assessed with the Clinical-Administered PTSD Scale for Children and Adolescents (CAPS-CA); (2) reporting a history of (repeated) physical and/or sexual abuse and/or domestic violence by a caretaker, family member, or person in authority; (3) the availability of a non-offending adult caregiver for the treatment, as the presence of a caregiver is part of the treatment design; (4) motivation and ability of the participant and the caregiver to attend weekly treatment sessions; (5) safe living circumstances to minimize the risk of retraumatization during the study; and (6) participants and caregivers have sufficient command of the Dutch language to participate in the treatment.

Exclusion criteria are (1) acute suicidal behavior or suicidal ideations requiring immediate hospitalization; (2) severe head trauma indicated by a score < 9 on the Glasgow Coma Scale as known from the participant’s medical history, to avoid brain dysfunction or retrograde amnesia of the traumatic event due to head injury; (3) concurrent psychotherapy during the study; (4) current severe mental disorder in the participant’s main caregiver (as evaluated by the responsible clinician), such as psychosis, severe episode of depression, or severe substance abuse, to assure the ability of the caregiver to participate in the treatment; (5) a sibling of the participant already participating in the study (to avoid the transference of treatment effects if siblings are randomized in different conditions); and (6) intellectual disabilities (IQ < 70).

### Randomization

Once the informed consent form is signed and the baseline questionnaires (T0 and T1) are completed, the randomization procedure begins. Participants are randomly assigned to either a control or an experimental group with a 1:1 allocation according to a computer-generated randomization schedule stratified by sex (male or female) and psychiatric diagnoses and using permuted blocks of random sizes. The block sizes will not be disclosed to ensure concealment. Participants will be randomized using randomization.com, an online randomization tool. Allocation concealment will be ensured because the person who performs randomization has no other role in the study. This person will prepare the randomization lists and seal the envelopes. The randomization code will not be released until the participant is recruited into the trial, which occurs after all baseline measurements have been completed.

To ensure allocation concealment, the steps involved in randomization, outcome measurements, and treatment are separated. Participants who meet the inclusion criteria and provide consent for participation will be randomized. Research assistants responsible for recruitment and outcome measurement will request randomization.

### Procedures

The recruitment of participants began in 2018 at Karakter, with a break in 2020 due to COVID-19. The recruitment is ongoing and is expected to be completed by 2024. All adolescents referred to Karakter sent a brief screening questionnaire called the Child Trauma Screening Questionnaire (CTSQ) [21]. This self-report questionnaire asks about traumatic life events, such as domestic violence, sexual abuse, or physical abuse. The research team screens the results from the CTSQ. If multiple traumatic life events are reported and the adolescent shows elevated scores on questions related to post-traumatic stress, the involved healthcare provider is contacted. The healthcare provider is informed about the MARS study and asked to consider participation in the study. The inclusion and exclusion criteria are verified by the medical staff involved with the participants. With the agreement of the healthcare provider and parents/legal guardians, the adolescent is invited to participate in a clinical structured interview (CAPS-CA).

After the CAPS-CA interview, the diagnostic results are discussed with the research team and treatment team of the MARS-study. A conclusion is drawn regarding whether the inclusion criteria for PTSD due to multiple interpersonal traumas are met. An advisory consultation will be scheduled with the participants and parents/guardians if the inclusion criteria are met. During this consultation, the participant is informed about their participation in the treatment study, informed consent is reviewed, and an information sheet about the study is provided. After the advisory consultation, the adolescents and parents/guardians have two weeks to decide whether to participate in the study. If the adolescents and their parents/guardians agree to participate, the randomization procedure is initiated, and a treatment provider is assigned within three weeks. The MARS-study requires written informed consent from both parents and adolescents. In addition, general practitioners are informed through postal letters.

### Interventions

#### Skills training in affective and interpersonal regulation (TRAP; dutch version of STAIR-A)

In Phase I of the phase-based treatment, we use an adapted version of the Skills Training in Affective and Interpersonal Regulation for Adolescents [12]. The purpose of this treatment is to address problems with affect and interpersonal regulation (as they negatively impact daily life) and to effectively utilize trauma-focused treatment. The program consists of twelve sessions, with different topics (e.g., distress tolerance, different kinds of role-plays, labelling and identifying feelings, enhancing adequate coping strategies, training self-soothing skills, etc.). All TRAP sessions have an identical format and structure: psycho-education about the rationale and goals of interventions, skills acquisition, skills application, and practice.

#### EMDR therapy

The protocol for EMDR therapy is an evidence-based trauma treatment aimed at resolving symptoms resulting from disturbing or unprocessed life experiences [22]. Treatment starts with recalling the traumatic memory and selecting the most disturbing part of this memory with associated dysfunctional thoughts and feelings. While concentrating on the traumatic memory, the participant’s working memory is taxed by employing eye movements for approximately 30 s. Repeatedly, the participant is asked to report what comes to mind, which may be cognitive, emotional, somatic, or imagistic experiences. After some sets of eye movements, the participant is asked to report a Subjective Unit of Disturbances (SUD) between 0 and 10, until the disturbance related to the memory reaches an SUD of zero and positive beliefs are rated strong on a VoC (Validity of Cognition, between 1 and 7). A wide array of studies support the working memory account as a mechanism that explains treatment effects. Recalling a traumatic episode depends on limited working memory resources. If a second task, taxing working memory, is executed during the recall of traumatic memory, fewer resources will be available to recall the traumatic episode. By performing both tasks simultaneously, the memory becomes less vivid and emotional and is stored in this new way. Consequently, negative cognitions lose credibility, and positive cognitions become more credible [23].

### Measurements

The study parameters are listed in Table 1. In order to collect data to answer the research questions, it will take the adolescent each time (T1, (T2), T3, T4) about 90–120 min to complete the interview and questionnaires. The total estimated burden for parents/legal guardians to complete the questionnaires will be approximately 120 min. Participants allocated to the TRAP + EMDR condition will undergo a mid-treatment assessment (T2) to investigate the effect of TRAP on both the primary and secondary outcomes.

**Table 1:** Study parameters

	Instrument	Informant	Nr. Items	**TRAP + EMDR**					**EMDR only**				
				T0	T1	T2	T3	ES	T0	T1	T2	T3	ES
Screening	CTSQ	Adolescent	24 items	x					x				
*Baseline*													
Demographic variables	Intake Questionnaire	Medical files			x					x			
Dissociative Experiences	A-DES	Adolescent	30 items		x					x			
*Primary outcome*													
PTSD-assessment	CAPS-CA	Adolescent	interview	x	x	x	x		x	x		x	
*Secondary outcome*													
Interpersonal problems	ECR-RC-mother	Adolescent	12 items	x	x	x	x		x	x		x	
Interpersonal problems	ECR-RC-father	Adolescent	12 items	x	x	x	x		x	x		x	
Emotion regulation	DERS	Adolescent		x	x	x	x		x	x		x	
Self Esteem	RSES	Adolescent	10 items	x	x	x	x		x	x		x	
Anxiety and Mood	RCADS	Adolescent	47 items	x	x	x	x		x	x		x	
Posttraumatic cognitions	CPTCI	Adolescent	25 items	x	x	x	x		x	x		x	
Behavior problems	CBCL	Medical files	113 items	x					x				
Behavior problems	Y-OQ-30	Parent	30 items	x			x		x			x	
Parental Stress	OBVL	Parent	34 items	x			x		x			x	
*Other measurements*													
Adherence		Medical files						x					x
Monitoring PTSD	CRIES-13	Adolescent	13 items					x					x
Patient-therapist relationship	SRS/ORS	Adolescent	8 items					x					x

*T0* screening, *T1* baseline, *T2* Mid treatment, *T3* post-treatment, *T4* 6-month follow-up, *ES* each session

### Screening for traumatic experiences

#### The child trauma screening questionnaire

The Child Trauma Screening Questionnaire (CTSQ) [21] is a self-reported measure that contains a 14 items list of traumatic life events and a 10 items list to index post-traumatic stress symptoms of re-experiencing and hyperarousal. Each life event can be answered with yes (scored as 1) or no (scored as 0). Scores > 4 indicate positive screening for trauma symptoms. The CTSQ has been shown to have good convergent validity [21]. Internal consistency was reported, with a Cronbach’s alpha of 0.69.

### Primary outcome

#### The clinician-administered ptsd scale for children and adolescents

The Clinician-Administered PTSD Scale for Children and Adolescents (CAPS-CA) [24] is a structured clinical interview used to establish the diagnostic status of the DSM-IV and DSM-V criteria for PTSD. The interviewer can rate the frequency and intensity of each symptom on a five-point Likert scale. Furthermore, each symptom can be rated as present or absent, as proposed by Weathers, Ruscio, and Keane [25] to score a symptom as being present. The CAPS-CA can be reliably administered by different interviewers. The Dutch CAPS-CA showed as good internal consistency, inter-rater reliability, convergent and divergent validity, and concurrent validity as the original English version [26]. The CAPS-CA will be administered three times (T1, T3, and T4) for adolescents in the EMDR-only group and four times in the TRAP + EMDR group (T1, T2, T3, and T4). Adding the CAPS-CA at T2 in the TRAP + EMDR condition will provide information about the effects and necessity of skills training TRAP as a standalone treatment.

### Baseline

#### The adolescent dissociative experience scale

The Adolescent Dissociative Experiences Scale (A-DES) [27] is a self-report scale used to measure the frequency of dissociative experiences among adolescents. The answers to the 30 items are marked on an 11-point scale ranging from 0 (never) to 10 (always). The total A-DES score is the mean of all item scores (range, 0–10). A mean score of 4.0 (Armstrong et al., 1997) is a commonly used cut-off for pathological dissociation. The A-DES has four theoretically derived subscales: amnesia, depersonalization/derealization, absorption, imaginative involvement, and passive influence. The A-DES has good internal consistency and test–retest stability and has proven to be valid across different cultural settings. The A-DES will be administered at T1.

#### Demographics

Demographic information of the participants will be collected from medical files and/or intake questionnaires. The gathered information will consist of age, sex, family structure, diagnoses of the child, school functioning, and socioeconomic status (SES) of the family.

### Secondary outcomes

#### The experiences in close relationships scale-revised child version

The Experiences in Close Relationships Scale-Revised Child version (ECR-RC) [28] is a self-report questionnaire on parent–child attachment that consists of 12 statements about the adolescents’ mother or father. Using a scale from 1 (not at all) to 7 (very much), six items tap into attachment anxiety (e.g., ‘I worry that my father/mother does not really love me’), and six items tap into attachment avoidance (e.g., ‘I prefer not to tell my father/mother how I feel deep down’). The reliability and validity of the ECR-R–C have been demonstrated in several independent samples. In terms of reliability, the ECR-R–C showed high levels of internal consistency, and in terms of validity, the ECR-R–C subscales correlated with depressive symptoms, emotion regulation strategies, and parenting dimensions [29, 30].

#### Difficulties in emotion regulation scale

Emotion dysregulation will be measured using the Difficulties in Emotion Regulation Scale (DERS) [31]. The DERS is a 36-item self-report questionnaire that measures six domains of emotion regulation difficulties: non-acceptance of negative emotions, difficulties in engaging in goal-directed behaviors, difficulties in accessing effective emotion regulation strategies, impulsivity, limited emotional awareness, and limited emotional clarity. The items were rated from 1 (almost never) to 5 (almost always). Higher DERS scores indicate greater emotion regulation difficulties. The DERS is a valid and reliable instrument for assessing emotional dysregulation [31, 32].

#### Rosenberg self-esteem scale

The Rosenberg Self-Esteem Scale (RSES) [33] will be used to assess self-esteem. It is a widely used 10-item Likert-type scale that measures self-esteem. Items are answered on a 4-point scale, from strongly agree to strongly disagree, measuring positive and negative feelings towards the self. The Dutch version of the RSES is a one-dimensional scale with high internal consistency and congruent validity, and a Cronbach’s alpha of 0.89 [33].

#### Revised child anxiety and depression scale

The Revised Child Anxiety and Depression Scale (RCADS) is a 47-item youth self-report questionnaire [34] with subscales including separation anxiety disorder (SAD), social phobia (SP), generalized anxiety disorder (GAD), panic disorder (PD), obsessive–compulsive disorder (OCD), and major depressive disorder (MDD). It also yields a Total Anxiety Scale (sum of the five anxiety subscales) and a Total Internalizing Scale (sum of all six subscales). Items are rated on a 4-point Likert scale ranging from 0 (“never”) to 3 (“always”). Additionally, the Revised Child Anxiety and Depression Scale – Parent Version (RCADS-P) similarly assesses parental reports of youth symptoms of anxiety and depression across the same six subscales.

#### Child post-traumatic cognitions inventory

The Child Posttraumatic Cognitions Inventory (CPTC) [35] is a self-report questionnaire that measures trauma-related cognition in children and adolescents. The questionnaire consists of 25 items that can be answered on a four-point Likert scale (range from 1 (strongly disagree) to 4 (strongly agree). The English version of the CPTCI has been validated for children and adolescents aged 6–18 years. The Dutch CPTCI has good reliability and validity [36], high internal consistency (Cronbach's 0.86–0.93), and good convergent validity.

#### Child behavior checklist

The Dutch parent-report version of the Child Behavior Checklist 6–18 years (CBCL) assesses a wide range of children's emotional and behavioral problems, aiming to identify children at a high risk of psychiatric disorders. The CBCL/6–18 comprises 120 items assessing behavioral and emotional problems. These items are answered on a 3-point Likert-type scale (0 = not true, 1 = somewhat or sometimes true, 2 = very true or often true) by parents. The scores display eight problem scales: withdrawn (1), somatic (2), anxious (3), social (4), thought (5), attention (6), rule-breaking (7), aggressive (8), and other problems. The sum of problem scales 1, 2, and 3 forms the scale ‘internalizing behavior’; 7 and 8 form ‘externalizing behavior.’ All subscales comprise the total problem scale. Some items contribute to more than one problem scale. The CBCL assesses a broad array of potential trauma-related symptoms, including those not captured by a PTSD-specific measure. T-scores are computed from raw scores; higher scores on the syndrome scale indicate greater severity of problems. A T-score of 63 (90th percentile) demarcates the clinical range, indicating that the child requires professional assistance. The CBCL/6–18 has well-established psychometric properties in clinical, non-clinical, and cross-cultural populations [37].

#### Youth outcome questionnaire

To assess the therapy outcome in terms of changes in symptom level, we will use the Dutch translation of the Youth Outcome Questionnaire [38]. The Y-OQ-30 has 30 items and can be completed in 10–15 min on a 5-point Likert scale with a range of 0 (never) to 4 (always). The Y-OQ-30 has six subscales: Somatic complaints, Social Isolation, Aggression, Behaviour problems, Hyperactivity, and Depression/ Anxiety. The Y-OQ-30 is a valid and reliable test for assessing changes [39].

#### Parental stress questionnaire

The OBVL (“Opvoedingsvragenlijst”) is a 34-item parent report questionnaire that measures experienced parental stress [40]. The questions are answered on a 4-point scale (1 = does not apply, 2 = applies a little, 3 = applies fairly, and 4 = applies completely). Scores on the subscale of problems in the parent–child relationship range from 6 to 24, where a score of 14 or higher indicates severe problems, for which treatment is indicated. Scores on ‘parenting problems’ range from 7 to 28, with a score of 18 or higher indicating severe problems [41]. The OBVL demonstrated good reliability and validity.

### Other measurements

#### Children's revised impact of event scale

The Children's Revised Impact of Event Scale (CRIES-13) [42] is a brief self-report questionnaire designed to screen for PTSD in children aged 8 years and older. It consists of 13 questions assessing posttraumatic intrusions, avoidance, and arousal. Children rated the frequency with which they had experienced each item during the past week using a four-point Likert scale (0 = not at all, 1 = rarely, 3 = sometimes, 5 = often). Psychometric properties have been previously reported [42], showing that the CRIES-13 is a valid measure of posttraumatic stress. In this study, the internal consistency of the CRIES-13 was 0.89. In this study, the CRIES-13 will be used to measure posttraumatic stress symptoms between each session during the course of treatment.

#### Outcome rating scale and session rating scale

To collect client feedback, we will use two brief questionnaires, the Outcome Rating Scale (ORS) and Session Rating Scale (SRS), which can be easily administered on a regular basis during treatment [43]. This allows treatment sessions to be evaluated at any time to ascertain whether individual treatments are ‘on the right track’ to a successful outcome. The ORS is primarily focused on the client’s well-being and is administered at the beginning of the treatment session. The SRS is administered at the end of the session and deals with how the client experienced the treatment session. The outcomes of the questionnaires are reflected in a graph per interview to allow the height of the score and progress to be visualized during the sessions.

### Sample size and power

This study implemented a non-inferiority trial. A non-inferiority trial is a type of clinical research study that aims to demonstrate that a (new) treatment is not worse than an existing treatment by a prespecified margin. Because the non-inferiority margin for CAPS-CA-5 scores had not yet been determined at the start of this study, we used the article published by Sloan et al. [44] to estimate the non-inferiority margin. The primary outcome was the total score on the Clinician-Administrated PTSD Scale for DSM-5 (CAPS-5). Non-inferiority was defined as a score of 10 points. However, the results showed that during follow-up, a non-inferiority margin of five points would have been sufficient to yield the same conclusion. Therefore, based on the article by Sloan et al. [44] and clinical reasoning, the largest clinically acceptable effect to declare non-inferiority is a change in the CAPS-CA-5 score of five points (d). Based on the study by Sloan and colleagues, the true mean difference (μ_B_-μ_A_) between the treatments is -1.82 (after 12 weeks). In a non-published pilot study of 17 adolescents, we calculated a mean total score on the CAPS-CA-5 of 38.2 with a standard deviation of 10.4 (σ). An equal number of participants will be included in both arms of the trial (r = 1).

For the estimation of the sample size (for 80 percent power and type 1 error of 2,5 percent) the following formula is used [45].

$${n}_{A}=\frac{7.85{\sigma }^{2}\left(r+1\right)}{{\left(\left({\mu }_{B}-{\mu }_{A}\right)-d\right)}^{2}r}$$

According to this formula, 36,5 participants per arm are required. As in the previous sample size calculation, we used the baseline variables as covariates. Based on Borm et al. [46, 47] we estimate that the design factor will be equal to 0.75, because we assume a correlation of 0.5 between the two measurements. Hence, we would need 36.5*0.75 = 27.4 participants per group. To compensate for an unknown clustering effect and unknown psychometric properties, and given the need to compensate for an expected dropout rate, we plan to include 40% more participants (76 participants in total), so that 38 participants will be recruited per arm.

### Data collection and management

Confidentiality is maintained throughout the current study. The handling of subjects’ personal data is in accordance with the European General Data Protection Regulation (in Dutch: Algemene Verordening Gegevensbescherming, AVG). To maintain anonymity of all data, participants will only be identifiable by a unique code assigned to the data of their inclusion. The code list will be digitally stored on the secured drive of Karakter, which is password protected and is only accessible to researchers involved in the project. Non-anonymous data (e.g., informed consent documents) will be digitalized and stored in password-secured folders that provide restricted access. All local databases will be secured with password-protected access systems. The online electronic data capture software CASTOR EDC will be used to collect and store the questionnaire data. All paper documents are stored in a locker at Karakter Child and Adolescent Psychiatry. Access to this storage is accessible to only a select few researchers.

### Statistical analysis

Data will be analyzed using SPSS V.29.0 for Windows (SPSS Incorporated). Pretreatment group differences with respect to age, sex, ethnicity, and type of traumatic event will be assessed using independent-sample t-tests for continuous data and χ^2^ tests for categorical data. Before testing, we will check all the data according to the appropriate assumptions.

To answer the primary question, a repeated-measures ANCOVA will be conducted. Baseline scores will be included as covariates, time as a categorical variable, and treatment condition as a fixed effect. The intercept will be treated as a random effect. We will examine the residuals to assess the model assumptions and goodness-of-fit. *P-values* will be reported to four decimal places, with *P-values* less than 0.001 reported as *p* < 0.001. For all tests, we will use 2-sided *P-values* with alpha =  < 0.05 level of significance. As this is a non-inferiority trial, both intention-to-treat and per-protocol analyses will be performed.

To evaluate the secondary outcomes, a repeated-measures ANCOVA will be conducted for the total score on the ECR-RC, DERS, RSES, RCADS, CPTCI, CBCL, OBVL, and Y-OQ-30. Baseline scores will be included as covariates, time as a categorical variable, and treatment condition as a fixed effect. The intercept will be treated as a random effect. We examine the residual to assess the model assumptions and goodness-of-fit. Delta-scores for pre- to post-treatment (T1 and T3) will be calculated, and an independent sample t-test will be used to compare the TRAP + EMDR and EMDR-only conditions. For the calculation of effect sizes (Cohen’s *d*) for within-group effects, we will divide the difference in means between pre- and post-treatment by the standard deviation of the difference in means. *P-values* will be reported to four decimal places, with *P-values* less than 0.001 reported as p < 0.001. For all tests, we will use 2-sided *P-values* with alpha =  < 0.05 level of significance.

To determine PTSD symptom changes on the CRIES-13, we will analyze the data for all time points in a linear mixed model. Time, treatment condition, the interaction term time × treatment condition, and sex will be entered as fixed factors, and age as a covariate in the model. The impact of missing data on outcome measurements will be evaluated using different methods, such as Last Observation Carry Forward (LOCF) and Multiple Imputation (MI).

### Adverse events

Adverse events (AE) are defined as any undesirable experience occurring to a subject during the study, whether considered related to undergoing dietary treatment. All adverse events observed by the researchers or reported by the participants will be recorded and processed according to legislation in The Netherlands. A serious adverse event (SAE) is an event that 1) results in death, 2) is life-threatening (at the time of the event), 3) requires hospitalization or prolongation of existing inpatient hospitalization, 4) results in persistent or significant disability or incapacity, 5) is a congenital anomaly or birth defect, and 6) may jeopardize the participant or may require an intervention to prevent one of the outcomes listed previously. All SAEs will be communicated to the coordinating investigator (first author), who will be responsible for reporting this information through the web portal ToetsingOnline to the accredited MREC that approved the protocol. Reporting of SAEs that are life-threatening or result in death will be reported no later than seven days after the first knowledge of SAEs in a preliminary report. The final report will be completed no later than eight days after the preliminary report. All AEs will be monitored until they diminish or reach a stable state. Follow-up may require 1) medical procedures, 2) additional tests, or 3) referral to a general physician or medical specialist. SAEs need to be reported until the end of the study in the Netherlands, as defined in the protocol.

## Discussion

The present paper describes the rationale, study design, and methods of the MARS-study: an RCT evaluating the effects of a phase-based treatment model compared to purely trauma-focused therapy in young individuals diagnosed with PTSD and on the possible symptoms of complex PTSD resulting from (repeated) sexual and/or physical abuse during childhood.

This study had several strengths. First, it sought to assess the effectiveness and efficacy of two widely utilized treatment models in a group of patients who are often overlooked in research owing to their complex symptomatology. To the best of our knowledge, an efficacy study of adolescents who have experienced (repeated) sexual and/or physical abuse during childhood is unprecedented. Second, although a phase-based trauma-focused model has recently been studied in adults (15–17), no similar study has been conducted in adolescents. Research on the effectiveness of trauma treatment in adolescents (symptoms of complex) PTSD is limited [20]. To our knowledge, this is the first randomized controlled trial to investigate and compare the effects of regulation skill training with those of a direct trauma-focused treatment approach in this population. Third, adding a follow-up period allows us to track the long-term effects of both treatments, which can provide valuable information about the sustainability of the treatment effects and whether there are any delayed or long-term benefits (sleeper effects) or side effects. Therefore, we can make more reliable and comprehensive conclusions regarding the effectiveness of and differences between interventions. Additionally, by adding a mid-treatment measurement, it is possible to investigate the effects of TRAP and better assess the added value of this phase-based treatment approach.

Although the proposed study has strengths, it is important to acknowledge several methodological issues that should be taken into consideration. First, the participants included in the study are those who have developed PTSD as a result of surviving (repeated) sexual and/or physical abuse during childhood. While our goal is to include individuals with Complex PTSD, the current study faces challenges due to the lack of reliable and validated instruments specifically designed to assess the complex manifestations of symptoms associated with complex trauma. Consequently, the study relies on trauma history as the primary criterion for inclusion rather than the comprehensive manifestation of complex trauma symptoms. However, as part of the secondary analyses, separate questionnaires are administered to assess domains related to Complex PTSD, including emotion regulation, interpersonal regulation, and negative self-image. Another important consideration pertains to the operationalization of the phase-based model. The concept of a 'phase-based model' can be interpreted differently, depending on various perspectives. In clinical settings, it encompasses more than one treatment protocol that focuses on skill development in regulation. For some clinicians, preparing patients for trauma-focused treatment requires a personalized approach that involves comprehensive psychoeducation, establishing a therapeutic alliance, and engaging with family members and trusted individuals. Training in regulation skills itself demands substantial time and effort from both the patient and therapist. However, it is crucial to note that the effectiveness of this approach has not been extensively studied, raising a limitation in our understanding of its impact and outcomes.

Finally, this study implemented a non-inferiority trial. A non-inferiority trial is a type of clinical research study that aims to demonstrate that a new treatment is not worse than an existing treatment by a prespecified margin. One benefit of this type of trial is that it allows for the evaluation of new treatments that may have advantages over existing treatments, such as fewer side effects or lower costs, even if they are not necessarily more effective. However, non-inferiority trials have limitations. One limitation is that they require a well-defined and clinically meaningful non-inferiority margin, which is challenging to determine. In addition, non-inferiority trials may be less likely to detect small but clinically meaningful differences between treatments, which could limit their ability to provide definitive conclusions regarding the relative effectiveness of different treatments.

## Conclusion

Treating adolescents with (symptoms of complex) PTSD stemming from (repeated) sexual and/or physical abuse during childhood is an immensely significant endeavor. However, there is a lack of consensus among trauma experts regarding the most effective approach. The current study is specifically designed to address this gap by evaluating the effectiveness of the proposed protocol. Through this research, we aim to generate additional and novel knowledge regarding trauma treatment. The findings of this study hold potential for important implications, offering valuable insights into the field of trauma treatment for severely traumatized adolescents.

## Data Availability

Data sharing is not applicable to this article, as no datasets were generated or analyzed during the current study. The final dataset will be available (anonymized) to other researchers at the end of the study. We will assess whether the aim of using the dataset is in conflict with our planned future publications. Otherwise, the dataset can be shared with other researchers.

## Abbreviations

A-DES: Adolescent Dissociative Experience Scale
AE: Adverse Event
CBCL: Child Behavior Checklist
CAPS-CA: Clinician-Administered PTSD Scale for Children and Adolescents
CCMO: Committee on Research involving Human Subjects
CRIES-13: Children’s Revised Impact of Event Scale
CPTCI: Child Post-Traumatic Cognitions Inventory
CTSQ: Child Trauma Screening Questionnaire
DERS: Difficulties in Emotion Regulation Scale
DSM-IV: Diagnostic and Statistical Manual of Mental Disorders (Fourth Edition)
DSM-5: Diagnostic and Statistical Manual of Mental Disorders, Fifth Edition
ECR-RC: Experiences in Close Relationships Scale-Revised Child version
EMDR: Eye Movement Desensitization and Reprocessing
ICD: International Classification of Diseases
ISTSS: International Society for Traumatic Stress Studies
MI: Multiple Imputations
NICE: National Institute for Health and Clinical Excellence
RCT: Randomized Controlled Trial
OBVL: Opvoedingbelastingvragenlijst
ORS: Outcome Rating Scale
PTSD: Post Traumatic Stress Disorder
RCADS: Revised Child Anxiety and Depression Scale
RSES: Rosenberg Self-Esteem Scale
SAE: Serious Adverse Event
SES: Socioeconomic Status
SRB: Safety Review Board
SRS: Session Rating Scale
TF-CBT: Trauma-Focused Cognitive Behavioral Therapy
TRAP: Training in affectieve en interpersoonlijke Regulatievaardigheden voor meervoudig getraumatiseerde Adolescenten Protocol
STAIR: Skill Training in Affective and Interpersonal Regulation, World Health Organization
WMO: Medical Research Involving Human Subjects Act
Y-OQ-30: Youth Outcome Questionnaire
